# Neutrophil-to-lymphocyte ratio and platelet-to-lymphocyte ratio in evaluation of inflammation in non-dialysis patients with end-stage renal disease (ESRD)

**DOI:** 10.1186/s12882-020-02174-0

**Published:** 2020-11-25

**Authors:** Peiyuan Li, Chenqi Xia, Peng Liu, Zhong Peng, Hong Huang, Juan Wu, Zhangxiu He

**Affiliations:** 1 Department of Gastroenterology, The First Affiliated Hospital of University of South China, Hengyang, 421001 PR China; 2 Department of Nephrology, The First Affiliated Hospital of University of South China, Hengyang, Hunan Province 421001 PR China; 3 Institute of Clinical Medicine, The First Affiliated Hospital of University of South China, Hengyang, 421001 PR China

**Keywords:** Neutrophil-to-lymphocyte ratio (NLR), Platelet-to-lymphocyte ratio (PLR), Inflammation, Non-dialysis patients with end-stage renal disease (ESRD)

## Abstract

**Background:**

Neutrophil-to-lymphocyte ratio (NLR) and platelet-to-lymphocyte ratio (PLR) have been reported to be associated with inflammation in end-stage renal disease (ESRD) receiving dialysis. However, the value of NLR and PLR in non-dialysis patients with ESRD remains unclear.

**Methods:**

Among 611 non-dialysis patients with ESRD in The First Affiliated Hospital of University of South China (2012–2018), we compared NLR and PLR in patients with high-sensitivity C-reactive protein (hs-CRP) levels of ≤3 mg/L vs. > 3 mg/L. Correlation of NLR and PLR to hs-CRP, PCT, ferritin were analyzed. Receiver operating characteristics (ROC) analysis was used for estimating sensitivity and specificity of NLR and PLR.

**Results:**

NLR was higher in the patients with high hs-CRP levels (> 3 mg/L), compared to patients with low hs-CRP levels (≤ 3 mg/L) [5.74 (3.54–9.01) vs. 3.96 (2.86–5.85), *p* < 0.0001]. Additionally, PLR was higher in high hs-CRP group than in low group [175.28 (116.67–252.26) vs. 140.65 (110.51–235.17), *p* = 0.022]. In the current study, NLR and PLR were both positively correlated with hs-CRP (rs = 0.377, *p* = 0.000 for NLR; rs = 0.161, *p* = 0.001 for PLR), PCT, leukocytes, neutrophils, platelets, and age. NLR or PLR with a cut-off value of 5.07 or 163.80 indicated sensitivity and specificity were 65.67 and 66.37% (AUC = 0.69) or 57.21 and 57.52% (AUC = 0.55), respectively.

**Conclusions:**

NLR or PLR was positively correlated with hs-CRP in non-dialysis patients with ESRD. NLR might be better for identifying inflammation than PLR in this population.

## Background

Inflammation is involved in the process of end-stage renal disease (ESRD) mainly caused by diabetic nephropathy and chronic glomerulonephritis [1–3]. Our previous study and others showed that inflammatory marker is a significant predictor of intima-media thickness (IMT) progression and increased IMT had poor survival in ESRD patients [4–6]. It has been reported that increased IMT, as a strong predictor of cardiovascular disease and mortality, was associated with inflammation even in non-dialysis patients [5, 7]. Thus, it is important to pay attention to inflammation in ESRD as well as non-dialysis patients.

Inflammatory markers such as C-reactive protein (CRP), procalcitonin (PCT), and ferritin are widely used in ESRD [8–10]. However, those traditional biomarkers have their limitations. The predictive value of CRP is rather nonspecific in dissecting a cause because multiple factors contribute to the inflammation of uremia [10]. PCT, a calcitonin precursor peptide, is a sensitive and specific indicator for infection, but its measurement is costly or inaccessible [11]. Ferritin has relative low accuracy in evaluating inflammation in ESRD since ESRD patients who had received intravenous iron also show higher ferritin level [9]. Therefore, a simpler, more convenient and useful marker is desired for use in clinical practice.

Recently, neutrophil-to-lymphocyte ratio (NLR) was reported to be associated with inflammation in ESRD including both hemodialysis (HD) and peritoneal dialysis (PD) patients [11–13], and estimate survival in HD patients [14, 15]. Studies suggested that platelet-to-lymphocyte ratio (PLR) was linked to inflammation and could predict mortality among HD patients [11, 14]. Both NLR and PLR are inexpensive, convenient, and have been widely served as prognostic indicators in several cancers such as esophageal or prostate cancer [16, 17]. Their application for evaluating inflammation in ESRD dialysis patients has been addressed. However, the value of NLR and PLR in non-dialysis patients with ESRD (those patients are in a special transition period that ESRD patients have to live through before dialysis or a kidney transplantation) remains unclear.

Therefore, in the current study, we studied a 7-year cohort of non-dialysis ESRD patients and sought to determine the relationship of NLR and PLR with inflammation in those patients.

## Methods

### Study population

A total of 611 non-dialysis patients (Age: 56.91 ± 14.62 y, 61.20% for males) with ESRD, admitted to The First Affiliated Hospital of University of South China from February 2012 to June 2018, were enrolled in this cross-sectional study. They were diagnosed as ESRD for the first time and these patients were evaluated by two nephrologists before starting dialysis. Patients were excluded if they had one of the following diseases: 1) a history of major surgery and inflammatory disease within the preceding 3 month; 2) end stage liver disease; 3) metastatic malignancies; 4) malabsorption syndromes. Their data was collected before the first dialysis.

### Study parameters

Data on patient demographics (age, gender), etiology of ESRD, blood biochemistry and inflammatory markers including high-sensitivity C-reactive protein (hs-CRP) (mg/L), PCT (ng/ml), and ferritin (ng/ml), complete blood count, NLR, and PLR were recorded in all patients. GFR (ml/min per 1.73 m^2^) = 186 × Scr^-1.154^ × age^-0.203^ × 0.742 (if female) × 1.233 (if Chinese), as described [18, 19]. Hs-CRP was set for low risk (< 1.0 mg/L), average risk (1.0–3.0 mg/L), and high risk (> 3.0 mg/L) as described before [11, 13]. In this regard, recorded data was also compared in patients with hs-CRP levels of ≤3 mg/L vs. > 3 mg/L in the present study. Correlation of NLR and PLR to age (years), serum levels of albumin (g/L), hs-CRP (mg/L), ferritin (ng/ml), PCT (ng/ml), leukocytes (10^9^/L), neutrophils (10^9^/L), lymphocytes (10^9^/L), and platelets (10^9^/L) were also studied.

### Laboratory analysis

Blood samples were drawn from all the individuals using uniform techniques after an overnight fasting period. Complete blood count and all biochemical analyses including serum creatinine, serum albumin, calcium, phosphorus, parathormone and ferritin were performed by automated procedures. The serum level of hs-CRP was measured by nephelemetric method (Roche, Hitachi Cobas C system, Mannheim, Germany). NLR was calculated as ratio of neutrophil to-lymphocyte counts and similarly PLR was calculated as the ratio of the platelet-to-lymphocyte count. Both were obtained from the same blood sample. All laboratory values were measured in the laboratory of The First Affiliated Hospital of University of South China by standardized methods.

### Statistical analysis

Numerical variables were tested for normal distribution with the Kolmogorov–Smirnov test. Normally distributed variables were summarized as mean (± SD) and compared using Student’s t-test. Non-normally distributed variables were summarized as medians with interquartile ranges (IQRs) and Mann–Whitney U test was conducted. Frequencies were provided for all nominal values and Fisher’s exact test was used for comparison of qualitative data. Spearman tests were used for correlation analysis. The use of NLR and PLR to accurately diagnose inflammation in ESRD patients without dialysis was evaluated by receiver operating characteristics (ROC) analysis. A sensitivity and specificity calculation for NLR and PLR was derived from the hs-CRP cut offs. *P*-value < 0.05 was considered as significant. All statistical analyses were carried out using SPSS 20.0 software (SPSS Inc., Chicago, IL, USA).

## Results

### Baseline characteristics of patients

The average age of the patients was 56.91, and 61.20% of them were males. Diabetic nephropathy (32.08%), chronic glomerulonephritis (29.30%), and hypertensive nephropathy (16.53%) were the leading etiological factors during the development of ESRD. Data on laboratory findings and inflammatory markers are presented in Table 1**.**

**Table 1:** Demographic and clinic characteristics and laboratory findings in non-dialysis ESRD patients

Demographics	
Age (y), mean ± SD	56.91 ± 14.62
Sex (male, %)	374 (61.20%)
**Etiology of ESRD, n (%)**	
Diabetic nephropathy	196 (32.08%)
Hypertensive nephropathy	101 (16.53%)
Chronic glomerulonephritis	179 (29.30%)
Other	87 (14.24%)
Undetermined	48 (7.85%)
**Blood biochemistry**	
Urea (mmol/L)	25.75 ± 11.53
Creatinine (umol/L)	805.01 ± 386.83
GFR (ml/min per1.73 m^2^)	7.83 (5.91–10.55)
Hemoglobin (g/L)	75.38 ± 19.09
Calcium (mg/dL)	7.80 ± 1.32
Phosphorus (mg/dL)	5.86 ± 2.36
Ca × P (mg^2^/dL^2^)	45.00 ± 17.87
Intact Parathormone (pg/mL)	271.34 (156.12–419.53)
Albumin (g/L)	36.13 ± 6.22
Ferritin (ng/mL)	296 (151.10–479.00)
Leukocytes (10^9^/L)	7.09 ± 3.19
Neutrophils (10^9^/L)	5.39 ± 2.98
Lymphocytes (10^9^/L)	1.08 ± 0.52
Platelets (10^9^/L)	170.18 ± 74.10
**Inflammatory markers**	
hs-CRP (mg/L)	6.87 (1.90–27.70)
PCT (ng/ml)	0.61 (0.26–1.96)
Neutrophil-lymphocyte ratio	4.48 (3.05–7.32)
Platelet-lymphocyte ratio	154.80 (109.98–235.47)
**Comorbidity, n (%)**	
Heart failure	136 (22.26%)
Coronary artery disease	115 (18.82%)
Cerebrovascular disease	13 (2.13%)
**Drug use within 1 month, n (%)**	
Steroid	3 (0.49%)
Cyclophosphamide	1 (0.16%)
Cyclosporine	1 (0.16%)

Data are expressed as mean ± SD, percentage or median (IQR). *ESRD* End-stage renal disease, *Ca* Calcium, *P* Phosphorus, *hs-CRP* High sensitivity C-reactive protein, *PCT* Procalcitonin

### Study parameters with respect to hs-CRP groups

Of 611 patients, 218 patients had low hs-CRP levels (≤ 3 mg/L), while 393 patients had high hs-CRP levels (> 3 mg/L). When compared to patients with lower (≤ 3 mg/L) hs-CRP levels, patients with high hs-CRP levels were determined to have significantly higher values for NLR [5.74 (3.54–9.01) vs. 3.96 (2.86–5.85), *p* < 0.0001], PLR [175.28 (116.67–252.26) vs. 140.65 (110.51–235.17), *p* = 0.022], ferritin [344.30 (192.70–586.88) vs. 217.60 (100.75–376.80) ng/ml, *p* < 0.0001], and PCT [0.69(0.33–2.03) vs. 0.42 (0.19–0.80), *p* = 0.034] **(**Table 2**).**

**Table 2:** Demographic and clinical characteristics and laboratory findings according to hs-CRP groups in non-dialysis ESRD patients

	hs-CRP ≤ 3 mg/L (*n* = 218)	hs-CRP > 3 mg/L (*n* = 393)	P
**Demographics**			
Age (year), mean ± SD	55.44 ± 13.55	58.96 ± 14.91	0.273
Sex (male, %)	124 (56.88%)	259 (65.90%)	0.029
**Etiology of ESRD, n (%)**			
Diabetic nephropathy	71 (32.57%)	125 (31.81%)	0.857
Hypertensive nephropathy	36 (16.51%)	65 (16.54%)	0.993
Chronic glomerulonephritis	65 (29.82%)	114 (29.01%)	0.853
Other	28 (12.84%)	59 (15.01%)	0.546
Undetermined	18 (8.26%)	30 (7.63%)	0.875
**Blood biochemistry**			
Urea (mmol/L)	24.58 ± 10.87	27.55 ± 12.37	0.068
Creatinine (umol/L)	775.84 ± 343.25	845.91 ± 434.48	0.083
eGFR (ml/min per1.73 m^2^)	7.99 (6.33–10.07)	7.82 (5.36–10.42)	0.175
Hemoglobin (g/L)	74.30 ± 18.95	75.52 ± 19.96	0.581
Calcium (mg/dL)	7.97 ± 1.16	7.72 ± 1.32	0.326
Phosphorus (mg/dL)	5.60 ± 2.31	6.04 ± 2.45	0.161
Ca × P (mg^2^/dL^2^)	43.89 ± 17.10	45.98 ± 18.49	0.053
Intact Parathormone (pg/mL)	269.87 (175.06–420.21)	265.18 (138.12–410.00)	0.370
Albumin (g/L)	36.85 ± 6.08	35.31 ± 6.26	0.569
Ferritin (ng/mL)	217.60 (100.75–376.80)	344.30 (192.70–586.88)	< 0.0001
Leukocytes (10^9^/L)	5.98 ± 2.27	8.01 ± 3.68	< 0.0001
Neutrophils (10^9^/L)	4.37 ± 2.01	6.28 ± 3.51	< 0.0001
Lymphocytes (10^9^/L)	1.08 ± 0.53	1.04 ± 0.53	0.613
Platelets (10^9^/L)	161.70 ± 69.64	175.48 ± 74.11	0.721
**Inflammatory markers**			
hs-CRP (mg/L)	1.04 (0.445–2.02)	18.20 (7.4–54.07)	< 0.0001
PCT (ng/ml)	0.42 (0.19–0.80)	0.69 (0.33–2.03)	0.034
Neutrophil-lymphocyte ratio	3.96 (2.86–5.85)	5.74 (3.54–9.01)	< 0.0001
Platelet-lymphocyte ratio	140.65 (110.51–235.17)	175.28 (116.67–252.26)	0.022
**Comorbidity, n (%)**			
Heart failure	46 (21.10%)	90 (22.90%)	0.685
Coronary artery disease	49 (22.48%)	66 (16.79%)	0.105
Cerebrovascular disease	6 (2.75%)	7 (1.78%)	0.559
**Drug use within 1 month, n (%)**			
Steroid	2 (0.92%)	1 (0.25%)	0.604
Cyclophosphamide	1 (0.46%)	0 (0.00%)	0.151
Cyclosporine	1 (0.46%)	0 (0.00%)	0.151

Data are expressed as mean ± SD, percentage or median (IQR). *ESRD* End-stage renal disease, *Ca* Calcium, *P* Phosphorus, *hs-CRP* High sensitivity C-reactive protein, *PCT* Procalcitonin

### Correlation analysis

**Fig. 1:**
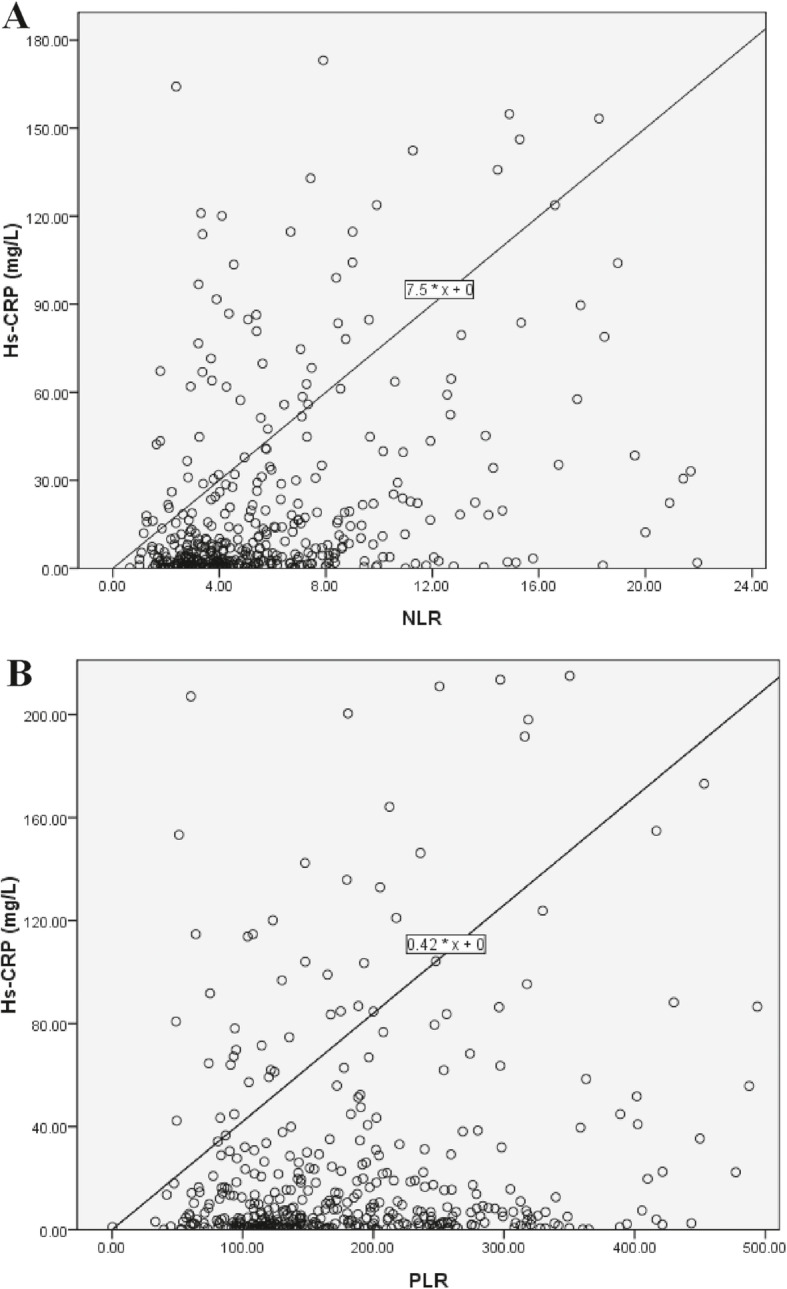
Correlation of (**a**) NLR and (**b**) PLR to hs-CRP in non-dialysis ESRD patients. NLR = Neutrophil-to-lymphocyte ratio; PLR = platelet-to-lymphocyte ratio; hs-CRP = high sensitivity C-reactive protein; ESRD = end-stage renal disease

Bivariate correlation analysis revealed that both NLR and PLR were positively correlated with hs-CRP (rs = 0.377, *p* < 0.0001 for NLR and rs = 0.161, *p* < 0.0001 for PLR) and PCT (rs = 0.285, *p* < 0.0001 for NLR; rs = 0.158, *p* = 0.037 for PLR). NLR was statistically positively correlated with ferritin (rs = 0.140, *p* = 0.003), while PLR has no relationship with ferritin (rs = − 0.039, *p* = 0.413) **(**Table 3**)**. The relationships of NLR and PLR to hs-CRP are displayed Fig. 1 and Table 4. The optimal cut-off value of NLR was 5.07 and the cut-off value of PLR was 163.80. The sensitivity and specificity were 65.67 and 66.37% for NLR, while were 57.21, 57.52% for PLR, respectively. Positive and negative likelihood ratios were 1.95 and 0.52 for NLR, while 1.35 and 0.74 for PLR, respectively.

**Table 3:** Bivariate correlation analysis of NLR and PLR to other parameters in non-dialysis ESRD patients

	Neutrophil-to-lymphocyte ratio		Platelet-to-lymphocyte ratio	
	rs	P	rs	P
Age (years)	0.177	< 0.0001	0.171	< 0.0001
Sex	0.038	0.350	−0.002	0.960
Albumin (g/L)	−0.075	0.07	−0.060	0.150
hs-CRP (mg/L)	0.377	< 0.0001	0.161	0.001
Ferritin (ng/mL)	0.140	0.003	−0.039	0.413
PCT (ng/ml)	0.285	< 0.0001	0.158	0.037
Leukocytes (10^9^/L)	0.510	< 0.0001	0.108	0.008
Neutrophils (10^9^/L)	0.691	< 0.0001	0.234	< 0.0001
Lymphocytes (10^9^/L)	−0.612	< 0.0001	−0.561	< 0.0001
Platelets (10^9^/L)	0.100	0.015	0.583	< 0.0001
Hemoglobin (g/L)	−0.18	0.658	0.069	0.094

*NLR* Neutrophil-to-lymphocyte ratio, *PLR* Platelet-to-lymphocyte ratio, *ESRD* End-stage renal disease, *hs-CRP* High sensitivity C-reactive protein, *PCT* Procalcitonin

**Table 4:** The relationships of NLR and PLR to hs-CRP in non-dialysis ESRD patients

	Neutrophil-to-lymphocyte ratio	Platelet-to-lymphocyte ratio
AUC	0.69	0.55
Sensitivity (%)	65.67	57.21
Specificity (%)	66.37	57.52
Positive likelihood ratio	1.95	1.35
Negative likelihood ratio	0.52	0.74
Cut-off value	5.07	163.80
*P* value	< 0.0001	

*AUC* Area under the curve, *NLR* Neutrophil-to-lymphocyte ratio, *PLR* Platelet-to-lymphocyte ratio, *ESRD* End-stage renal disease, *hs-CRP* High sensitivity C-reactive protein

### ROC analysis of the relationship between NLR, PLR, and hs-CRP

**Fig. 2:**
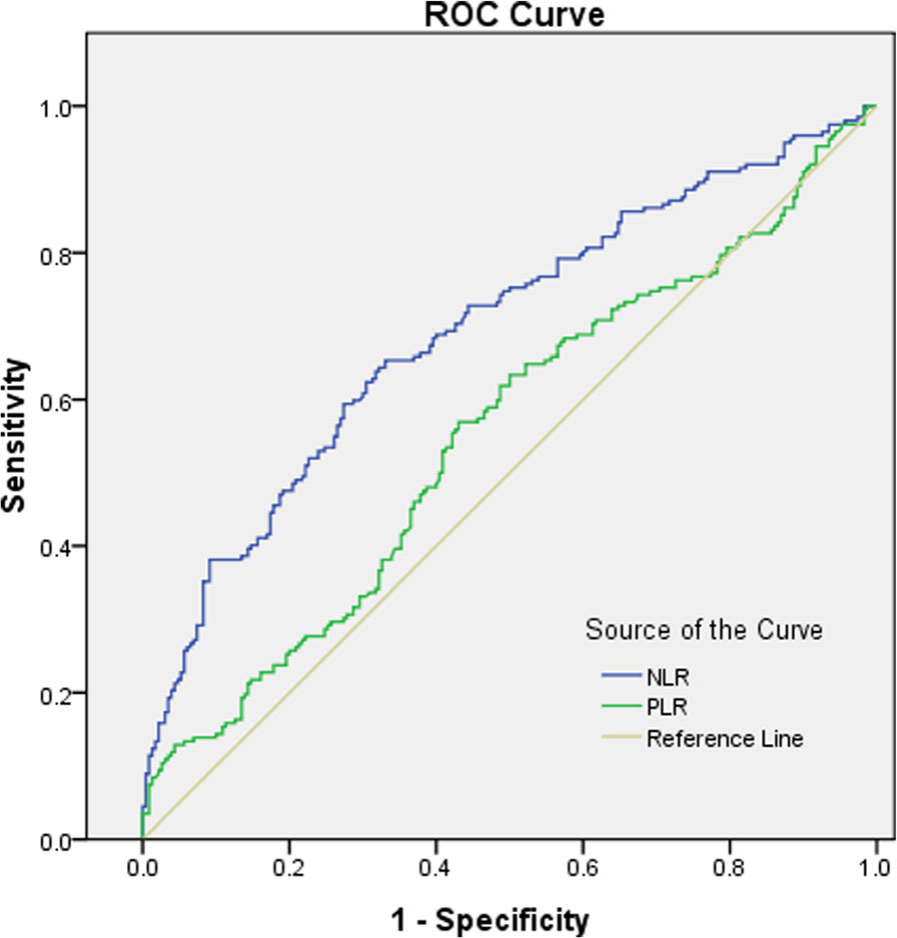
ROC analysis of the relationship among NLR, PLR and hs-CRP in non-dialysis ESRD patients. NLR = Neutrophil-to-lymphocyte ratio; PLR = platelet-to-lymphocyte ratio; hs-CRP = high sensitivity C-reactive protein; ESRD = end-stage renal disease

Figure 2 illustrates ROC curve which indicated poor sensitivity and specificity of PLR **(**Fig. 2**).** However, ROC curve of NLR (AUC = 0.69) showed significantly (*p* < 0.0001) larger area than PLR (AUC = 0.55) (Table 4).

## Discussion

Our findings in a cohort of non-dialysis patients with ESRD first revealed that higher (> 3 mg/L) hs-CRP levels were carrying with higher values for NLR and PLR in patients. Moreover, patients with higher hs-CRP levels tended to have significantly higher values for PCT, ferritin, leukocytes and neutrophils. Importantly, we explored that both NLR and PLR were positively correlated with hs-CRP and PCT.

Our analysis showed that higher values for NLR and PLR in high (> 3 mg/L) hs-CRP level group than those in low (≤ 3 mg/L) level group in non-dialysis ESRD patients, is consistent with the result in HD patients [11]. Similar to hs-CRP, PCT and ferritin are also inflammatory markers [8, 9]. Thus, it is reasonable to have the result that high hs-CRP level group has significantly higher values of PCT and ferritin compared to low hs-CRP level group in our current study. Additionally, we found that high hs-CRP level group had significantly higher values of leukocytes and neutrophils when compared to low hs-CRP level group. Our previous reports that CKD patient had increased level of activated leukocytes and neutrophils may partly explain this since inflammation can lead to activation and up-regulation of those cells [20–22].

Surprisingly, we found that non-dialysis ESRD patients had relatively lower median levels of hs-CRP (6.87 mg/L) than those (16.8 mg/L) in HD patients, but higher median levels of NLR (4.48) and PLR (154.80) in comparison to those (NLR = 3.4; PLR = 139) in HD patients respectively [11]. The trends of NLR, PLR, and hs-CRP are not parallel in ESRD patients without dialysis and HD patients. Uremia, endogenous factors as well as the dialysis procedure itself may be responsible for the high prevalence of inflammation and high level of hs-CRP in HD patients [23, 24]. It has been reported that NLR was found negatively correlated with glomerular filtration rate (GFR) and positively correlated CKD stage [25–27]. Therefore, non-dialysis ESRD patients from rural district with kidney disease in more advanced stage may account for the high level of NLR and PLR in our result [28].

NLR and PLR have previously been reported as inflammatory markers for patients with ESRD. One study demonstrated that NLR was positively correlated with tumor necrosis factor-α (TNF-α) in 61 ESRD patients receiving PD or HD for ≥6 months [12]. The study showed that the NLR was significantly higher in PD patients than HD patients. Another study compared NLR and PLR in 62 ESRD patients also receiving PD or HD for ≥6 months [13]. The study showed that ESRD patients with PLR ≥ 140 had significantly higher NLR, IL-6, and TNF-α levels when compared to patients with PLR < 139. The study also showed that PLR was found to be superior to NLR in terms of inflammation in ESRD patients. A recent study enrolled 100 ESRD patients on maintenance HD for ≥3 months [11]. The study revealed that the correlation relationships between the NLR or PLR and hs-CRP were statistically robust. In the present study, we recruited 611 non-dialysis patients with ESRD in a cross-sectional study. NLR and PLR were positively correlated with hs-CRP in non-dialysis ESRD patients, in line with other studies in PD or HD patients [11–13]. As to our knowledge, this is the first report about NLR and PLR in evaluation of inflammation in non-dialysis patients with ESRD. In addition, a strength of our study is that we included a wider range of patients.

We found that high hs-CRP level group was determined to have significantly higher values for ferritin positively correlated with NLR in non-dialysis ESRD patients, while the published papers suggested that there is no difference of ferritin value between high and low hs-CRP groups and no relationship between NLR and ferritin in HD patients [11]. Our data was different from previous reports. This may be attributed to commonly receiving intravenous iron to treat anemia in HD patients, while non-dialysis ESRD patients mostly receive oral iron poorly absorbed in advanced CKD, especially non-dialysis ESRD, leading to high levels of ferritin in HD patients and making the effect on the analysis in the population [11, 29–32]. Although PCT and TNF-α have both been shown to play a central role in the vicious circle of inflammation, PCT instead of TNF-α was analyzed statistically positively correlated with NLR or PLR in ESRD patients (11). One explanation is that PCT could serve as a better inflammatory marker than TNF-α in ESRD patients. This is supported by the reports that PCT level is more valuable indicator than TNF-α for predicting severity of acute pancreatitis, bacterial infection after bronchoscopy or in neutropenic febrile children with acute lymphoblastic leukemia [33–35].

NLR has proved to be a better marker than PLR or WBC as a predictor of survival in HD patients [14, 15]. Retrospectively, Catabay et al. compared the mortality predictability of NLR and PLR among 108,548 incident HD patients [14]. The authors found high NLR, but not PLR, in incident HD patients predicted mortality, especially in the short-term period. In another study, Ouellet et al. evaluated NLR as a predictor of survival in 5782 incident HD patients [15]. NLR is superior to total WBC count for prediction of all-cause mortality in incident and prevalent HD patients and was identifies as a novel and robust predictor of all-cause mortality in those patients. Moreover, NLR was demonstrated deterioration in renal function and assessed as a predictor of worsening renal function in CKD patients [25–27]. In the current study, our results elucidated that NLR or PLR was statistically correlated with hs-CRP in non-dialysis ESRD patients. However, it should also be noted that, the AUC for the ROC relationships was not enough to draw a conclusion that NLR or PLR was good substitute for hs-CRP. Taken together, NLR or PLR may be potential mixed markers of inflammation, renal function and mortality in CKD patients. In this regard, further study should be needed to validate the mixed role in CKD patients.

The present study has some limitations. First, our study is a single-center research. Second, the analyses were based on a single measurement of laboratory parameters that may not reflect the relation over time. Third, multivariable adjustment was not conducted (such as diabetes control) due to insufficient data. Finally, we mainly focused on hs-CRP, and its comparison to other inflammatory markers including albumin, IL-6, and TNF-α was limited. Thus, multi-center well-designed study is required to further clear relationship between inflammation and NLR or PLR, and whether the use of these ratios contributes clinically in non-dialysis ESRD patients.

## Conclusion

We first reported that NLR or PLR was positively correlated with hs-CRP in non-dialysis patients with ESRD and NLR might be better in identifying inflammation than PLR in this population.

## Data Availability

The datasets used and/or analyzed during the current study available from the corresponding author on reasonable request.

## Abbreviations

ESRD: End-stage renal disease
CKD: Chronic kidney disease
HD: Hemodialysis
CRP: C-reactive protein
PCT: Procalcitonin
NLR: Neutrophil-to-lymphocyte ratio
PD: Peritoneal dialysis
PLR: Platelet-to-lymphocyte ratio
hs-CRP: High-sensitivity C-reactive protein
WBCs: White blood cells
ROC: Receiver operating characteristics
GN: Glomerulonephritis
GFR: Glomerular filtration rate
TNF-α: Tumor necrosis factor-α
